# Synthesis and characterization of transition metal complexes of 4-Amino-5-pyridyl-4H-1,2,4-triazole-3-thiol

**DOI:** 10.1186/2193-1801-2-510

**Published:** 2013-10-05

**Authors:** Raghad Haddad, Emad Yousif, Ahmed Ahmed

**Affiliations:** Department of Chemistry, College of Science, Al-Nahrain University, Baghdad, Iraq

**Keywords:** Triazole ring, Triazole complexes, Transition metal complexes

## Abstract

Series of coordination complexes of Ni(II), Cu(II), Zn(II), Cd(II) and Sn(II) metal with 4-amino-5-(pyridyl)-4H-1,2,4-triazole-3-thiol, as a ligand has been successfully prepared in alcoholic medium. The prepared complexes were characterized quantitatively and qualitatively by using: microelemental analysis, FTIR spectroscopy, UV-visible spectroscopy, ^1^H and ^13^C NMR, magnetic susceptibility and conductivity measurements. This triazole ligand act as bidentate that coordination to the metal ions through sulphur and amine group. According to the spectral data of the complexes a tetrahedral geometry was suggested for these complexes except Cu (II) complexes which exhibit a square structure.

## Introduction

Heterocyclic chemistry has now become a separate field of chemistry with long history, present society and future prospects. Nitrogen, oxygen and sulfur are considered the most hetero atoms known. Heterocyclic compounds are considered one of an important type of organic compounds due to their implication in drugs and industrial studies (Carey 1980), (Acheson, 1989), (Zamani et al., 2004). Triazoles are five memberd heterocyclic compounds with molecular formula C_2_H_3_N_3_ containing three nitrogen and two carbon atoms (Siddiqui et al., 2011). There are two types of triazole, the 1,2,3-triazoles and the 1,2,4-triazoles (Bele and Singhvi, 2011), (Clayden et al., 2001), (Bruice, 1998). Amine and thione-substituted triazoles have been studied as anti-inflammatory and anti-microbial agents and other applications (Awad et al., 2008). Triazole are considered to be good coordinating ligands (Al-Maydama et al., 2008), because they involved both hard nitrogen and soft sulfur atom as thio amide group. This ligand have doner group that coordinate with wide range of metal ions (Narayana and Gajendragad, 1997). The tatumerisum form could occur in triazole (Davari et al., 2010). The potential coordinating sites are: (i) sulfur of thiol group, (ii) nitrogen of the primary amino group, (iii) two nitrogen atoms at position 1 and 2 in triazole ring system (Narayana and Gajendragad, 1997). This ligand contain an S = C^_^N^_^N unit that allow for bidentate coordination to metal ions through amine and thio substituted to form a stable five member ring (McCarrick et al., 2000). Thus this ligand is polydentate, it has been shown and experimentally verified that Complexes of polydentate ligands are called chelate complexes. They tend to be more stable than complexes derived from monodentate ligands (Hartwig 2010), Furthermore five or six membered chelate is by far the most common and the most stable. A new thio- Triazole complexes with selected metals was prepared (Majeed and Alabdeen, 2012). In this paper, the preparation and characterization of Cu(II), Ni(II), Zn(II), Cd(II) and Sn(II) complexes with 4-amino-5-(pyridyl)-4H-1,2,4-triazole-3-thiol are described.

## Materials and methods

All the reagents, starting materials as well as solvents were purchased commercially and used without any further purification. The melting points were recorded in Coslab melting point apparatus. Elemental C, H, N and S analysis were carried out on a Fison EA 1108 analyzer. The Infrared (FTIR) spectra were recorded by using FTIR.8300 Shimadzu spectrophotometer by using CsI disc in the frequency range of 4000–200 cm^-1^. The ultraviolet–visible (UV–VIS) spectra were recorded by using Shimadzu UV–VIS. 160 A-Ultra-violet spectrophotometer in the range of 200–1100 nm. The magnetic susceptibility values were obtained at room temperature using Magnetic Susceptibility Balance Johnson Matthey. Conductivity measurements were carried out by using WTW conductivity meter. Atomic absorption measurements were obtained by using Shimadzu 680 cc-flame. The spectra of ^1^H and ^13^C NMR spectra were recorded on a Bruker Ultrasheild 300 MHZ in Jordan, using deuterated DMSO-*d*6 as the solvent and tetramethylsilane, TMS as the internal standard.

### Synthesis of 4 amino-5-(pyridyl)-4H-1,2,4-triazole-3-thiol (ligand)

A mixture of isonicotinic acid (1 g) (0.0072 mol) and (0.44 g) (0.008 mol) from potassium hydroxide, was dissolved in (10 ml) ethanol. After mixture was dissolved then (2 ml) (0.014 mol) from carbon disulfide and was added slowly. The reaction mixture was stirred for 10 hours. Dry ether (10 ml) was added and the yellow precipitate was filtered, washed with ether and dried. The salt was obtained in almost quantitative yield and was employed to the next step. The yellow precipitate (potassium salt) was added to an excess of hydrazine hydride (20 ml), and was refluxed with stirring until the evaluation hydrogen sulfide; it was ceased by lead acetate paper. After cooling the reaction mixture was filtered, and then was acidified by Hydrochloric acid to yield the white precipitate (Siddiqui et al., 2010). Yield (62%), m.p. (210–212).

### Synthesis of this ligand's complexes

Ethanolic solution of the suitable metal salts [Cupper (II) acetate, Tine (II) Chloride, Zinc (II) acetate dihydride, Cadmium (II) acetate and Nickel (II) acetate] was added to an ethanolic solution of 4-amino-5-(pyridyl)-4H-1,2,4-triazole-3-thiol in 1:2 (metal:ligand) molar ratio and refluxed for two hours, crystalline colored precipitates was formed at room temperature. The resulting solids were washed by hot methanol and left to dried and recrystallized from ethanol (Majeed et al., 2004).

## Results and discussion

Melting points and physical properties of all the compounds studied are tabulated in Table 1. The data of CHNS and were obtained using flame atomic absorption technique. The calculated values were in a good agreement with the experimental values.

**Table 1:** **Physical data of prepared complexes**

Complexes	Color	M.P.	Elemental analysis theoretical (Experimental)				
			% C	% H	% N	% S	% M
**L**	White	212–214	45.91 (46.23)	5.30 (5.75)	33.47 (33.82)	15.32 (14.98)	- -
Ni(L)_2_ **1**	Green	240–242	40.01 (39.22)	5.25 (4.11)	29.16 (30.01)	13.35 (13.73)	12.22 (12.28)
Cu(L)_2_ **2**	Dark green	222–224	39.61 (40.21)	5.19 (5.89)	28.87 (29.32)	13.22 (13.58)	13.10 (16.02)
Zn(L)_2_ **3**	Off white	178–180	39.46 (40.05)	5.17 (6.13)	28.76 (29.30)	13.17 (13.52)	13.43 (17.28)
Cd(L)_2_ **4**	White	255–257	35.99 (40.25)	4.72 (5.25)	29.23 (28.88)	12.01 (12.52)	21.05 (23.85)
Sn(L)_2_ **5**	Yellow	230–232	35.57 (35.12)	4.66 (4.85)	25.93 (25.54)	21.97 (21.55)	21.97 (6.152)

The physical analytical data, melting point and elemental analysis of (L) and its complexes are tabulated in Table 1.

### Infra-red spectroscopy

**Figure 1:**
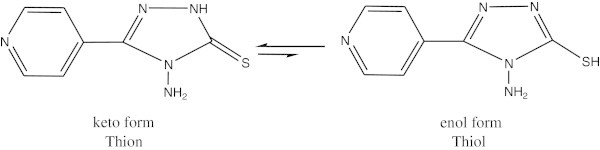
**Tatumerisum form in triazole.**

The FTIR spectrum of this ligand (L) showed some characteristic stretching bands at: 3250 and 3213, 2736, 1645, 673 assigned to NH_2_, S-H, C = N of triazole ring, and the last one is for stretching of C-S bond, respectively which could be found in complexes 1-5 (Pavia et al., 2001), (Silvertein and Bassler, 1980), (Majeed, 2010), (Yousif et al., 2005). The tatumerisum form could occur in triazole , see Figure 1. It is responsible to expect deprotonation of ligand molecule before complexation, the complete disappearance of the band due to ν(S-H) in the spectra of complexes unambiguously support this view. After deprotonation, the ligand can link with the metal ion at either by N or the S of thioamid group. Bonding at S is more favorable because such a thing would result in a stable five membered chelate (Narayana and Gajendragad (1997)).

The exceptional case is that the n(C = N) of complexes 1–5 were found to be shifted to a lower wavelength number compared to the ligand, L signifying that the coordination took place via the nitrogen atom of the ligand (L) (Sliverstein et al., 2005). The frequencies of NH_2_ bands were shift due to complexation. The band of S-H in the ligand was disappeared when complexation occur, but the bands of C-S also shifted to the higher frequency due to increasing of the bond order of carbon - sulfate bond result from complexation of the metal ion to the ligand through sulfate. And the band of C = N is shifted to the lower frequency due to complexation, but the other bands such as C = C, C-H aromatic were didn’t show any shifting because they aren’t participate in the complexation (Flifel and Kadhim, 2012). Another new bands was appeared which were supported by the appearance frequencies of M-S, M-N (Cheremisina et al., 1972), (Qurban, 2011), (Yousif et al., 2004). The major IR bands and their probable assignment are given in Table 2:

**Table 2:** **Key infrared data of L and complexes 1–5**

Complexes	NH_2_	-S-H	C = N	C-S	M-N	M-S
**L**	3250, 3213	2736	1645	673	-	-
**Ni(L)**_**2**_ **1**	3280, 3228	**-**	1643	690	530	459
**Cu(L)**_**2**_ **2**	3321, 3286	**-**	1620	694	532	428
**Zn(L)**_**2**_ **3**	3329, 3286	**-**	1640	694	529	432
**Cd(L)**_**2**_ **4**	3440, 3391	**-**	1639	693	528	451
**Sn(L)**_**2**_ **5**	3200, 3165	**-**	1643	690	528	432

### Nuclear magnetic resonance

The data of ^1^H NMR and ^13^C NMR of 4-amino-5-(pyridyl)-4H-1,2,4-triazole-3-thiol and its complexes displayed good solubility in DMSO. The proton nuclear magnetic resonance spectral data gave additional support for the composition of the complexes. The observed changes are evidences of complexation had happened because the chemical shift of a compound is heavily depended on its electronic environment. (Yousif et al., 2010), (Ibraheem et al., 2010), (Cos-kun, 2006).

### Ligand

**Figure 2:**
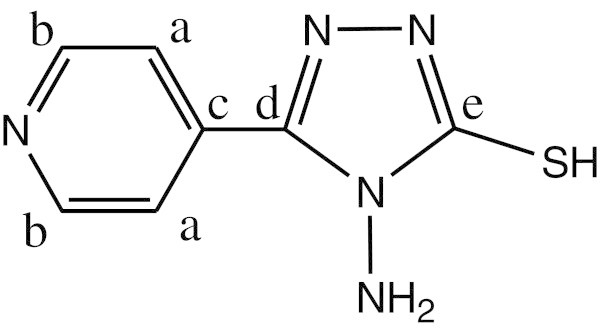
**The structure of ligand.**

^1^H NMR data (ppm), *δ*_H_(300 MHz, DMSO-d_6_) shows signals at 5.301 (2H, s, NH_2_), 8.014,8.025-8.744,8.755 (4H, d,d, CH aromatic ring) and 10.189 (1H, s, SH). ^13^C NMR shows chemical shift at 121.564 (carbon a), 150.133 (carbon b), 132.891 (carbon c), 147.308 (carbon d) and 167.551 (carbon e) (See Figure 2).

### Complex 1

**Figure 3:**
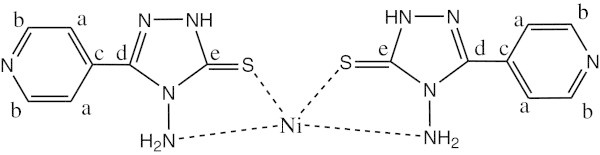
**The structure of complex 1 Ni(L)**_**2**_.

^1^H NMR data (ppm), *δ*_H_(300 MHz, DMSO-d_6_) shows the signals at 3.314 (2H, s, NH_2_) (this peak is shifted to lower field due to its attachment to the zinc atom), 7.945,8.014-8.739,8.678 (4H, d,d, CH aromatic ring) and 11.099 (1H, s, NH). ^13^C NMR shows chemical shift at 125.001 (carbon a), 149.583 (carbon b), 134.991 (carbon c), 155.683 (carbon d) and 183.548 (carbon e). (See Figure 3).

### Complex 3

**Figure 4:**
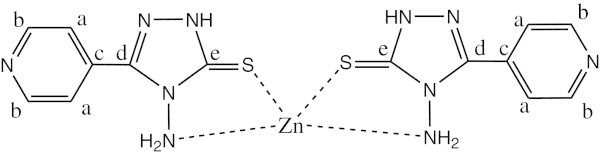
**The structure of complex 3 Zn(L)**_**2**_.

^1^H NMR data (ppm), *δ*_H_(300 MHz, DMSO-d_6_) shows the signals at 3.354 (2H, s, NH_2_) (this peak is shifted due to its attachment to the metal atom), 8.024-8.702 (4H, m, CH aromatic ring) and 10.012 (1H, s, NH). ^13^C NMR shows chemical shift at 121.705 (carbon a), 149.322 (carbon b), 134.035 (carbon c), 158.297 (carbon d) and 180.114 (carbon e). (See Figure 4).

### Complex 4

**Figure 5:**
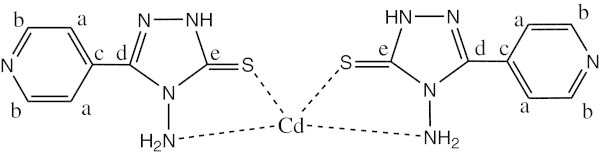
**The structure of complex 4 Cd(L)**_**2**_.

^1^H NMR data (ppm), *δ*_H_(300 MHz, DMSO-d_6_) shows the signals at 3.270 (2H, s, NH_2_) (for the reason mentioned above), 7.901,7.952-8,625,8.690 (4H, d,d, CH aromatic ring) and 11.101 (1H, s, NH). ^13^C NMR shows chemical shift at 120.992 (carbon a), 149.603 (carbon b), 134.036 (carbon c), 150.297 (carbon d) and 187.329 (carbon e). (See Figure 5).

Tables 3 and 4 show the ^1^H NMR, ^13^C NMR data of L and metal complexes 1,3 and 4 in DMSO-*d*6 Chemical shift, d (ppm).

**Table 3:** ^**1**^ **H NMR data of L and metal complexes 1,3 and 4 in DMSO-** ***d*** **6**

Complexes	C-H aromatic	NH_2_	S-H	N-H
**L**	(8.014,8.025-8.744,8.755)d,d	(5.301)s	(10.189)s	-
Ni(L)_2_ **1**	(7.945,8.014-8.739,8.678)d,d	(3.314)s	-	(11.099)s
Zn(L)_2_ **3**	(8.024-8.702)m	(3.354)s	-	(10.012)s
Cd(L)_2_ **4**	(7.901,7.952-8,625,8.690)d,d	(3.270)s	-	(11.101)s

Chemical shift, d (ppm).

**Table 4:** ^**13**^ **C NMR data of L and metal complexes 1,3 and 4 in DMSO-** ***d*** **6 chemical shift, d (ppm)**

Complexes	Carbon a	Carbon b	Carbon c	Carbon d	Carbon e
**L**	121.564	150.133	132.891	147.308	167.551
Ni(L)_2_ **1**	125.001	149.583	134.991	155.683	183.548
Zn(L)_2_ **3**	121.705	149.322	134.035	158.297	180.114
Cd(L)_2_ **4**	120.992	149.603	134.036	150.297	187.329

### Ultraviolet–visible spectroscopy

The absorption spectra of the ligand (L) and these complexes with the ligand (L) were recorded in DMSO solvent in range of 250–900 nm. The electronic spectra of (L) and its complexes was illustrate in Table 5. The electronic spectra of this ligand show 3 bands at (263, 302, 309) due to intraligand transition (π-π*), (π-π*), (n-π*) electronic transition, respectively. From Table 5, complexes 1–5 also showed the similar electronic transition but with shifting comparing with the ligand, (L). For complexes 1 and 2, the electronic transitions of the metal *d* orbitals (*d*-*d* electronic transition) observed in the Ni(II) and Cu(II) located in the visible region as extra information. In Ni(II), *d*-*d* electronic transition appeared at 620 nm assigned to the ^3^ T_1(F) →_^3^ T_1(P)_ and ^3^ T_1(F) →_^3^A_2(F)_ transition, respectively ( Greenwood and Earnshaw, 2002), (Hassan et al., 2008). For Cu(II), the bands appeared at 280, 300, 312 and 451 nm were attributed to (π-π*), (n-π*), charge transfer and ^2^ T_2_ → ^2^E_2_ respectively (Figgis, 2000), (Schönherr, 2004), (Majeed, 2008).

**Table 5:** **Electronic spectra of prepared compounds**

Complexes	Absorption	Transition
**L**	263, 302, 309	(π-π*), (π-π*), (n-π*)
**Ni(L)**_**2**_ **1**	262, , 610	(π-π*),^3^ T_1(F) →_^3^ T_1(P)_
**Cu(L)**_**2**_ **2**	280, 300, 312, 451	(π-π*), (n-π*), L → Cu(CT), ^2^ T_2_ → ^2^E_2_
**Zn(L)**_**2**_ **3**	264, 300, 310	(π-π*), (π-π*), (n-π*)
**Cd(L)**_**2**_ **4**	262, 310	(π-π*), (n-π*)
**Sn(L)**_**2**_ **5**	265, 310	(π-π*), (n-π*)

Where (*) symbol means the excited states.

But the other complexes (4, 5, 6), were diamagnetic as expected for d^10^ ions, so that no (d-d) transition can be expected in the visible region (Chohan, 2009).

### Magnetic susceptibility and conductivity measurements

Magnetic measurements are widely used in studying transition metal complexes. The magnetic property is due to the presence of unpaired of electrons in the partially filled d- orbital in the outer shell of these elements. The magnetic moment value of complex 1 was 1.09 B.M. and referred as paramagnetic. Complex 2 showed that the magnetic moment value is 0.7 B.M. and believed that the copper (II) metal moiety exhibited distorted square planar geometry (Win et al., 2011). Complexes 3–5 are diamagnetic and there were no magnetic moment recorded in this study (Chohan, 2009).

Conductivity measurement of these complexes was recorded as a solution in ethanol solvent. This measurement gives an idea if a solution is electrolyte or not. Table 6 show the molar conductivity measurements of complexes 1–5, it was shown that all the prepared complexes were found to be non-electrolyte (Majeed et al., 2010).

**Table 6:** **Conductivity measurement and magnetic moment of L and its complexes**

Complexes	Conductivity (μS/cm)	Megnetic moment (BM)
**L**	**-**	**-**
Ni(L)_2_ **1**	3	1.09
Cu(L)_2_ **2**	1	0.7
Zn(L)_2_ **3**	3.4	0
Cd(L)_2_ **4**	2	0
Sn(L)_2_ **5**	12	0

**Figure 6:**
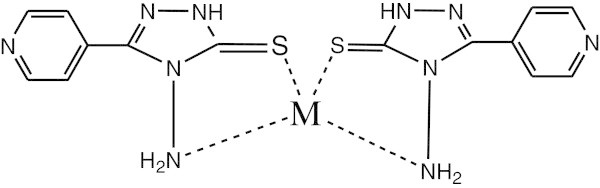
**The proposed structure of complexes 1–5 (where M = Ni(II), Cu(II), Zn(II), Cd(II) and Sn(II)).**

Based on the spectral study, complexes 1–5 exhibited distorted tetrahedral geometry except complex 2 (distorted square planar) (Foo et al., 2013). The proposed structure of complexes 1–5 is shown below. (See Figure 6).

## Conclusion

The ligand 4-amino-5-(pyridyl)-4H-1,2,4-triazole-3-thiol was successfully synthesized. The ligand was treated to different metal ions salts to afford the corresponding complexes. It concluded that the ligand coordinated through amino and thiol groups to the metal atom leading to the formation of five member ring chelate. Square planar geometry was proposed for the copper complex. The other complexes were proposed to be tetrahedral.
